# Improvements in Depression Outcomes Following a Digital Cognitive Behavioral Therapy Intervention in a Polychronic Population: Retrospective Study

**DOI:** 10.2196/38005

**Published:** 2022-07-05

**Authors:** Aarathi Venkatesan, Benjamin Forster, Prasanna Rao, Melissa Miller, Michael Scahill

**Affiliations:** 1 Vida Health San Francisco, CA United States

**Keywords:** depression, anxiety, CBT, digital mental health intervention, cognitive behavioral therapy, digital health, obesity, diabetes, mental health

## Abstract

**Background:**

Digital mental health interventions have shown promise in reducing barriers to effective care for depression. Depression and related mental disorders are known to be highly comorbid with common chronic physical conditions, such as obesity and type 2 diabetes. While some research has explored the interaction dynamics of treating populations living with both mental and physical disorders, very little is known about such dynamics in digital care.

**Objective:**

We aimed to examine the effectiveness of a 12-week, therapist-supported, app-based cognitive behavioral therapy program in improving symptoms of depression and anxiety. The studied population included adults with a heavy burden of chronic physical disease, including obesity and type 2 diabetes.

**Methods:**

A total of 1512 participants with at least moderate depression were enrolled. The treatment cohort consisted of 831 (54.96%) participants who completed a follow-up assessment. The program included structured lessons and tools (ie, exercises and practices) and offered one-on-one weekly video counseling sessions with a licensed therapist for 12 weeks and monthly sessions thereafter. The clinically validated 8-item Patient Health Questionnaire (PHQ-8) and the 7-item Generalized Anxiety Disorder scale (GAD-7) were used to assess depression and anxiety, respectively. Linear mixed-effects modeling was employed to examine changes in depression and anxiety over time. Given correlation among various measures of program usage, a composite variable for depth of usage was used to analyze the correlation between usage and changes in depressive symptoms. Body weight changes from baseline were assessed primarily with digitally connected scales.

**Results:**

Out of 831 participants in the treatment cohort, 74.5% (n=619) showed a clinically significant reduction in depressive symptom severity after 12 weeks, where follow-up PHQ-8 scores had shifted downward by at least one diagnostic category. In total, 67.5% (n=561) of the participants showed a reliable improvement in PHQ-8 scores as measured by the reliable change index. There was an average reduction of 5.9 (SD 5.2) points (*P*<.001) between baseline and follow-up. Greater program usage was correlated with greater likelihood of reliable improvement in depressive symptoms (odds ratio 1.3, 95% CI 1.1-1.5; *P*=.002). An exploratory analysis of body weight changes with a multilevel, mixed-effect model suggested that reliable improvement in depressive symptoms at follow-up was associated with significantly greater weight loss at 9 months (β=–1.11, *P*=.002).

**Conclusions:**

The results provide further support that digital interventions can support clinically meaningful improvements in depression. Some form of synergy in treatment of comorbid depression and obesity or diabetes could be studied in future research. The study was limited by postintervention participant attrition as well as the retrospective observational study design.

## Introduction

In 2016, mental health disorders affected more than 1 billion people worldwide [1]. Among mental health disorders, clinical depression carries a lifetime risk of 15% to 18% [2] and has the strongest association with disability-adjusted life years, a global benchmark of disease burden [1,2]. Depression is also the psychiatric condition most strongly associated with suicide [3]. The relationships among clinical depression, well-being, and health care costs have been well documented, with costs of care for patients living with depression higher across spending categories [4,5].

It has been estimated from nationally representative surveys that the prevalence of depressive symptoms in the United States increased 3-fold during the COVID-19 pandemic, with the majority of those affected already having been at elevated risk [6]. Simultaneously, the pandemic has accelerated the adoption of digital technology to deliver health care, particularly for mental health, and highlighted the promise of remotely delivered health services when traditional models may not have been available or accessible [7].

Digital mental health interventions (DMHIs) are potentially scalable and effective treatment solutions for mental health. Although research evaluating the effectiveness of DMHIs notes significant heterogeneity in terms of study design, clinical model, and intervention characteristics, meta-analytic reviews of smartphone-based interventions have observed a clinically significant treatment effect compared to both wait-list and active controls for depression and anxiety symptoms [8-10].

There is considerable research consensus that cognitive behavioral therapy (CBT) is a highly effective therapeutic modality for the treatment of clinical depression and anxiety because of relatively short intervention durations with strong outcomes [11,12]. Specifically, CBT-based digital interventions were associated with a significantly greater reduction in depression compared to non-CBT therapeutic approaches (eg, mindfulness, psychoeducation, and mood monitoring) [9]. In the context of DMHIs, digital CBT interventions appear to be equivalent to face-to-face interventions in terms of treatment efficacy [13-16]. Furthermore, a recent assessment of digital CBT offerings that evaluated patient preference, cost savings, and clinical benefit concluded that a provider-guided digital CBT program was likely the most effective approach for depression and anxiety compared to self-guided and face-to-face interventions [17].

In addition to evaluating the efficacy of CBT-based digital interventions, there has been growing research interest in exploring their mechanisms, particularly in the role of program engagement in DMHIs. Recently, Chien et al [18] employed a machine learning–based approach to explore patterns of engagement with a digital CBT program and their relationship with improvements in depression and anxiety. While the study did identify five distinct classes of program engagement, from low to high engagement, all classes were all positively associated with clinical improvement. Higher engagement classes were associated with greater improvement in depressive symptoms, so a possible dose-response effect was suggested.

However, it has been observed that user engagement can be difficult to define and measure [19]. Where usage generally measures observable actions, engagement implies some subjective experience of the digital intervention with a focus on the quality of the experience [20,21]. Furthermore, as Torous et al [22] note, usage over time, a commonly used measure of engagement, cannot distinguish a user whose mental health needs have been met by an app with a single instance of usage from one who needs repeated access for support.

One app feature that has been previously operationalized as a measure of DMHI engagement is lesson completion [23]. Lessons are typically brief pieces of evidence-based content intended to support a skill or health habit. They can also serve a crucial function in mental health interventions, digital or otherwise. CBT, in particular, emphasizes the practice of skills in between sessions by way of “homework assignments” or lessons in order to reinforce practices such as cognitive reappraisal [24]. Kazantzis et al [25] observed a stronger treatment effect size for CBT-based therapeutic interventions that incorporated homework when compared to interventions without a homework component. Lesson completion has also been used as a measure of treatment adherence and has shown a positive association with improvements in depression and anxiety [17,25-29]. It has been noted that the ubiquity of smartphones and the flexibility of the technology enables access to extensive content, on demand or needs based, that is difficult to replicate in traditional delivery models [28,30].

Lastly, an often-overlooked dimension of clinical depression, particularly in the context of DMHIs, is its frequent co-occurrence with chronic health conditions, such as type 2 diabetes and obesity [31-33]. Among other aspects, depression is known to hamper self-care and medication adherence [33]. Cross-sectional research suggests a 1.18 times greater likelihood of depressive symptoms in individuals with obesity than in those without [34]. Similarly, the risk of prediabetes and related measures appears to be markedly elevated among those newly diagnosed with depression [35,36]. This relationship tends to be stronger among women [34,35]. In their meta-analytic review of collaborative care to treat depression and diabetes in tandem, Atlantis et al [31] observed significant improvements in both depression and glycemic management with tandem treatment. Another systematic review showed that psychological interventions tailored for people with diabetes were effective in improving both glycemic management (ie, hemoglobin A_1c_ [HbA_1c_]) and elevated diabetes distress [37]. There are indications that the relationship between chronic conditions and depression are bidirectional, with an increased incidence of diabetes among those with diagnosed depression [38-40]. Many app-based health interventions have a singular health focus (eg, depression, weight, or diabetes management), making it more challenging to fully understand the impact and the influence of co-occurring conditions on treatment adherence and health outcomes [41].

In sum, research suggests that CBT-based DMHIs are feasible and effective solutions for depression and anxiety disorders [42-44]. There are some preliminary indications that the extent of program usage may influence treatment adherence and outcomes [45]. There remain open research questions on how intervention type, mode of delivery, and mechanisms may affect treatment outcomes. There has also been less focus on DMHIs in populations living with co-occurring depression and chronic health conditions.

In this study, we evaluated the Vida CBT Program for moderate depression and anxiety in a polychronic adult population, that is, one with a high prevalence of chronic health conditions. Vida Health is a Health Insurance Portability and Accountability Act–compliant, app-based platform for management of both mental and physical health that combines tailored content with counseling by licensed therapists and other health education specialists. The platform is available directly to consumers or as a benefit from select employers and health plans. The primary objective of this study was to assess changes in depression following a 12-week digital CBT program: the Vida CBT Program. We hypothesized that participants who completed the program would show a reduction in depressive symptoms and that measures of program usage would be positively associated with these improvements. As part of a preliminary exploratory analysis, we also evaluated changes in weight among participants concurrently enrolled in a Vida physical health program. We suspected that improvements in depression would be positively associated with stronger weight loss.

## Methods

### Study Design

This study used a single-arm, retrospective design to evaluate changes in depression and anxiety following the Vida CBT Program.

### Ethics Approval

The study protocol and informed consent statement was reviewed and approved by the Western Institutional Review Board Inc (protocol No. 20192591), an independent institutional review board. All data were fully anonymized prior to data analysis. Informed consent statements were sent to participants upon enrollment in the Vida CBT Program.

### Measures

The 8-item Patient Health Questionnaire (PHQ-8) was used to assess severity of depressive symptoms. The following standard scoring cutoffs were applied to classify depressive symptom severity: 0 to 4 (asymptomatic or minimal), 5 to 9 (mild), 10 to 14 (moderate), 15 to 19 (moderately severe), and 20 or higher (severe). Anxiety symptoms were assessed using the 7-item Generalized Anxiety Disorder scale (GAD-7) using the following standard score cutoffs to classify anxiety symptom severity: 0 to 4 (asymptomatic or minimal), 5 to 10 (mild), 11 to 17 (moderate), and 18 or higher (severe). The PHQ-8 and GAD-7 are widely used in clinical settings and have shown robust reliability and validity [39,40]. The assessments were administered in the app automatically at program start, week 6, week 12, and every 3 months thereafter for up to 1 year. Participants were encouraged to complete the survey on the day of receipt but had the option to complete the assessment at any point during the 2 weeks following receipt, after which the survey would disappear until the next assessment time point. Therapists also had the option of sending the instrument to the participant at any point during the intervention as they deemed clinically appropriate.

Body weight was a secondary outcome measure in this study. Weight outcomes were either recorded via a connected wireless scale or self-reported by the participant using a logging tool available in the Vida Health app. Participants could either sync their personal wireless scale to the app or order a digitally connected scale via the app.

### Study Sample and Recruitment

The study was open to adults, 18 years of age or older, who were fluent in English and had access to a smartphone or tablet. Participants were recruited between September 2019 and January 2021 using a combination of emails, mailers, and phone outreach efforts. The Vida Health app is available to individuals across the United States and can be downloaded from the Apple App Store or Google Play. Participants were drawn from several insurance plans and employers for whom Vida Health was offered as a covered benefit.

Upon downloading the app, participants completed a brief intake questionnaire that included name, contact information, basic demographics (ie, age, gender, height, and weight), and existing health conditions. Participants were offered a variety of health domains to focus on. Some participants chose to begin the Vida CBT Program directly. Others began in the Vida digital programs for weight loss or diabetes management described previously [46,47]. Participants received and completed an intake PHQ-8, and those with a score of 10 or greater (moderate depression) were included in the study. All participants also completed a baseline GAD-7 assessment. Eligible participants were paired with a licensed therapist based on their state of residence and preferred times for consultations. Therapists were mental health professionals working for Vida Health, licensed by their state’s respective licensure board. Participants with severe depression (PHQ-8 score ≥20) or severe anxiety (GAD-7 score ≥15) were ineligible for the Vida CBT Program and were referred to alternative sources of care. Additional exclusion criteria included eating disorders, substance use disorder, suicidality, homicidality, acute posttraumatic stress disorder, and episodes of mania or psychosis. Participants who presented with any of the above symptoms during the intervention were referred for care outside of Vida Health. Participants who chose to enroll initially in weight loss or diabetes management programs and were then screened into the Vida CBT Program had the option to continue both programs simultaneously.

### Therapeutic Approach and Intervention

A fundamental focus of CBT is to address maladaptive thinking patterns by understanding the associations among thoughts, emotions, and behaviors [48]. As part of the Vida CBT Program, participants received structured multimedia (ie, audio, video, or text) lessons, activities, and practices within the app. Based on core CBT principles, such as guided discovery and conscious re-evaluation, these lessons were designed to increase awareness of one’s thinking patterns and support the practice of alternative, adaptive thoughts [24]. Ahead of their initial consultation with a Vida therapist, participants completed an informed consent form for psychotherapy that detailed their rights to confidentiality and limits to confidentiality, including mandated reporting requirements as stipulated by the therapist’s licensing board and state regulations. At the initial consultation, therapists performed a comprehensive biopsychosocial assessment that included a review of previous treatments and diagnoses, current presenting problem, and symptoms. Participants could communicate with their therapist using live video or audio consultations as well as with asynchronous messaging in the app.

**Figure 1:**
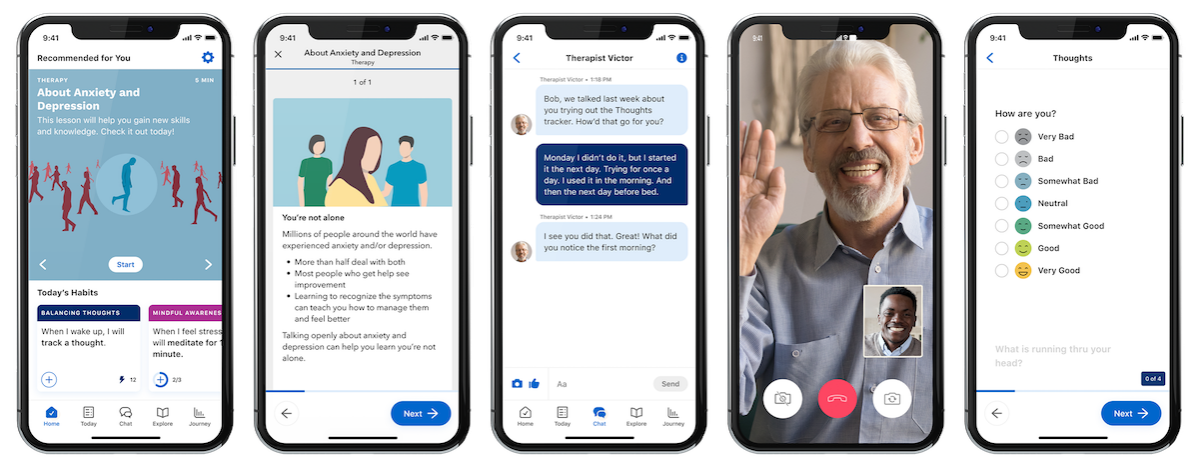
Screenshots from the Vida CBT Program. CBT: cognitive behavioral therapy.

Following the intake, therapists documented their initial diagnostic impressions and developed individualized treatment plans with short-term and long-term goals. Participants were offered weekly video or audio consultations with their therapist for the first 12 weeks and shorter monthly follow-up sessions thereafter for up to 1 year. Each therapist consultation comprised setting goals and homework activities for the upcoming week along with a review of strategies for further cultivating concepts and skills learned from earlier sessions. In between sessions, participants could complete assigned homework and use a thought tracker in the app. Lessons, once shared, remained available in the app for the participant to review and revisit concepts. The thought tracker feature allowed participants to record current thoughts using a Likert scale to assist in identifying, evaluating, and restructuring distorted thought patterns. In program weeks 10 through 12, therapists worked with participants to create a Wellness Recovery Action Plan intended to support maintenance of acquired skills and improved functioning and to prevent relapse [49]. Select screenshots from the program are shown in Figure 1.

Participants who were concurrently enrolled in a program for chronic disease management also worked with an additional Vida provider (ie, a certified health coach or registered dietitian) who could collaborate on care with the relevant therapist. As in the Vida CBT Program, they were offered synchronous consultations weekly for up to 12 weeks and monthly thereafter for up to 1 year. All providers received extensive training on motivational interviewing, an approach that leverages improving perceived self-efficacy and autonomy to facilitate healthy behavior change [50]. Additionally, participants received app content covering topics such as nutrition, exercise, and medication adherence. All content was informed by evidence-based research and literature on health behavior change as described previously [46,47,51].

### Statistical Plan

Change in depression, based on PHQ-8 scores, between baseline and follow-up was the primary dependent variable. We performed a paired, 2-tailed *t* test to assess if there was a significant change in PHQ-8 scores from baseline. We used a second paired *t* test to evaluate changes in anxiety scores among participants who scored in the moderate anxiety range at baseline (ie, GAD-7 score ≥11). Mean normalization was applied to all continuous predictors (eg, age). A Boolean variable was created for the presence of co-occurring anxiety (1 = baseline GAD-7 score ≥11). Gender was also coded as a binary variable (1 = female). Since we could not assume that all therapists were equally effective, all regression analyses were conducted using a cluster-robust approach with therapist as a cluster group variable [52]. The reliable change index (RCI) for depression and anxiety was also computed. RCI, a commonly used measure in psychometrics, is the ratio of the difference in pre-post assessment scores to the standard error of measurement [47,48]. An RCI score of 1.96 or higher (ie, 1 above the 95% CI) is regarded as an indication of reliable, statistically meaningful change [53].

To explore the interaction between program usage and changes in depression, we tabulated five program features that broadly encompass the program experience: number of therapist consultations, number of messages sent to the therapist, number of “core” lessons opened, number of thoughts logged, and total number of content pieces opened. Core lessons were those related to fundamental CBT concepts (eg, cognitive restructuring, behavioral activation, and techniques for addressing maladaptive thinking) [24]. Data exploration revealed a right skew for each of the usage factors, that is, a subpopulation of participants who used the app features quite extensively. In order to retain these heavy app users, but to limit their influence on downstream analyses, all usage factors were right-winsorized at the 99th percentile [54]. The treatment cohort showed a notable female predominance, a finding not unusual in studies of mental health service use in the United States [55-57]. In this study, in order to adjust for this, the established technique of oversampling was employed, wherein 715 participants were drawn at random from the 116 participants who did not identify as female [58,59].

Finding, as expected, that features of program usage were correlated, we constructed a composite variable to limit collinearity. Participants were assigned an ordinal variable from 0 to 5, where 1 unit was assigned for each usage feature in which the participant was above the 25th percentile. Thus, someone with a depth-of-usage score of 5 had activity above the 25th percentile in each of the usage factors, while a score of 0 indicated usage within the 25th percentile across all usage features. We chose the 25th percentile as it empirically separated users into high and low usage categories without selecting only for the right tail of usage as alluded above. With reliable change as the binary dependent variable (1 = reliable improvement in depression symptoms), a cluster-robust logistic regression evaluated the relationship depth of usage and improvement in depression symptoms. Controls included therapist cluster, baseline PHQ-8 score, gender, presence of anxiety, and age.

A supplementary analysis evaluating changes in weight outcomes among participants concurrently enrolled in the Vida weight loss program was conducted. Analysis was restricted to participants who had been enrolled for at least 6 months and had logged weight at least twice in that period. A linear, mixed-effects model was used to address potential heterogeneity in the frequency and number of weight logs by participants and provider-level differences [60]. Percent change in weight from baseline was regressed on the following fixed factors: program time (in months), baseline BMI category (obesity or overweight), and reliable change (1 = reliable improvement in PHQ-8 scores; 0 = no reliable improvement) [61]. Participants and providers were specified in the model as random factors.

All data preparation and analyses were performed using Python (version 3.7.9; Python Software Foundation) and Stata (version 16.1; StataCorp LLC).

## Results

### Overview

**Figure 2:**
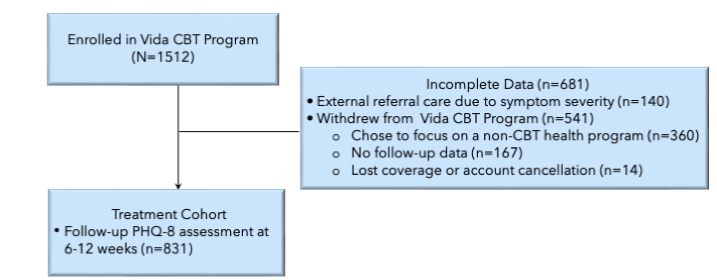
A schematic of the participant flow. CBT: cognitive behavioral therapy; PHQ-8: 8-item Patient Health Questionnaire.

A total of 1512 participants enrolled in the Vida CBT Program between September 2019 and January 2021. A schematic of the participant flow is presented in Figure 2. Of those, 54.96% (n=831) had a follow-up PHQ-8 assessment between weeks 6 and 12 and were considered part of the treatment cohort. Analyses evaluating changes in depression scores from baseline were restricted to the treatment cohort. The remainder of the study cohort, equal to 45.04% (n=681) of the participants, failed to complete a follow-up PHQ-8 during the assessment window. These participants were excluded from the primary analyses; however, we performed a supplementary intention-to-treat (ITT) analysis to evaluate overall changes in PHQ-8 scores from baseline across the entire cohort. Among those without a valid follow-up, 71.5% (487/681) did not have any assessment after their baseline. For these members, a baseline carryforward approach was employed. The remaining 28.5% (194/681) of the participants completed an assessment before finishing 6 program weeks. For these members, a last-value carryforward approach was applied.

Baseline characteristics of the study cohort are reported in Table 1. In addition to depression, the entire study cohort self-reported living with at least one chronic physical health condition (ie, type 2 diabetes, cardiovascular disease, or obesity). There were no significant differences in baseline PHQ-8 scores between the treatment and incomplete groups at baseline (*t*_1510_=1.2, *P*=.22). There was a significantly lower rate of comorbid anxiety, defined as a GAD-7 score of 11 or higher, among participants in the treatment cohort (*χ*^2^_1_=19.5, *P*<.001). A 2-tailed chi-square analysis indicated that there were significantly more women in the treatment cohort compared to the program noncompleter group (*χ*^2^_1_=7.1, *P*=.01). There were also more participants concurrently enrolled in a health coaching program for a chronic condition in the treatment cohort (*χ*^2^_1_=171.1, *P*<.001). Lastly, we observed a significant average difference in age of 2.3 years between the treatment cohort (mean 48.5, SD 11.4 years) and noncompleters (mean 46.2, SD 12.4 years; *t*_1510_=–3.8, *P*<.001).

**Table 1:** Demographic characteristics of the treatment and intention-to-treat study cohorts.

Characteristic			Incomplete data (n=681)		Treatment (n=831)		Total (N=1512)
Participants (N=1512), n (%)			681 (45.0)		831 (55.0)		1512 (100)
**Gender, n (%)**							
	Female	552 (81.1)		715 (86.0)		1267 (83.8)	
	Male	127 (18.7)		113 (13.6)		240 (15.9)	
	Not disclosed	2 (0.3)		3 (0.4)		5 (0.3)	
Age in years, mean (SD)			46.2 (12.4)		48.5 (11.4)		47.5 (11.9)
Baseline PHQ-8^a^ score, mean (SD)			14.6 (3.3)		14.4 (3.4)		14.5 (3.3)
Has anxiety (GAD-7^b^ score ≥11), n (%)			467 (68.6)		478 (57.5)		945 (62.5)
Enrolled in physical health program, n (%)			360 (52.9)		697 (83.9)		1057 (69.9)
**Chronic physical health condition, n (%)**							
	Obesity	554 (81.4)		723 (87.0)		1277 (84.5)	
	Cardiovascular disease	367 (53.9)		503 (60.5)		870 (57.5)	
	Diabetes	316 (46.4)		468 (56.3)		784 (51.9)	

^a^PHQ-8: 8-item Patient Health Questionnaire; depressive symptom severity by score is classified as follows: 0 to 4 (asymptomatic or minimal), 5 to 9 (mild), 10 to 14 (moderate), 15 to 19 (moderately severe), and 20 or higher (severe).

^b^GAD-7: 7-item Generalized Anxiety Disorder scale; a score of ≥11 indicates moderate to severe anxiety.

### Principal Results

Out of 831 participants in the treatment cohort, 74.5% (n=619) showed a clinically significant reduction in depressive symptom severity in 12 weeks, where follow-up PHQ-8 scores had shifted downward by at least one diagnostic category. A total of 67.5% (n=561) of the treatment cohort participants showed a reliable improvement in PHQ-8 scores as measured by the RCI. There was an average reduction of 5.9 (SD 5.2) points between baseline and follow-up (Table 2). A 2-tailed, paired *t* test revealed a significant reduction in scores between baseline and follow-up (*t*_830_=32.9, *P*<.001). A cluster-robust linear regression examining the association between change in PHQ-8 scores and baseline scores, co-occurrence of anxiety, gender, age, and concurrent enrollment in physical health revealed a significant inverse relationship between baseline and change in PHQ-8 scores (β=–1.7, *P*<.001). That is, greater baseline depression severity was, unsurprisingly, associated with greater reduction of depression at follow-up. We observed that the co-occurrence of anxiety was associated with a smaller reduction in depression scores (β=1.33, *P*<.001). In supplementary analyses, a 2-tailed, paired *t* test including the entire ITT cohort, using carryforward as above, indicated a slightly attenuated but still significant reduction in PHQ-8 scores at follow-up (mean –3.6, SD 4.99; *t*_1511_=29.1, *P*<.001).

Among the 478 participants with moderate anxiety or higher at baseline (ie, GAD-7 score ≥11), 89.7% (n=429) provided a follow-up assessment between weeks 6 and 12. A paired *t* test revealed a significant average reduction in anxiety scores of 6.1 (SD 5.4) points from baseline (*t*_428_=22.96, *P*<.001; Table 2). Additionally, 57.7% (n=276) of these participants had a reliable improvement in anxiety scores from baseline. A cluster-robust linear regression showed that higher baseline GAD-7 scores were associated with greater reductions in anxiety scores at follow-up (β=–3.5, *P*<.001). Age, gender, and baseline PHQ-8 scores were not significantly associated with anxiety score changes.

**Table 2:** Estimated marginal means of PHQ-8 and GAD-7 scores at baseline and follow-up.

Assessment type and time point			Score, estimated marginal mean (bootstrapped 95% CI)
**PHQ-8^a^**			
	Baseline	14.4 (14.2-14.6)	
	Follow-up (12 weeks)	8.5 (8.1-8.8)	
**GAD-7^b^**			
	Baseline	14.8 (14.5-15.0)	
	Follow-up (12 weeks)	8.8 (8.3-9.3)	

^a^PHQ-8: 8-item Patient Health Questionnaire; depressive symptom severity by score is classified as follows: 0 to 4 (asymptomatic or minimal), 5 to 9 (mild), 10 to 14 (moderate), 15 to 19 (moderately severe), and 20 or higher (severe).

^b^GAD-7: 7-item Generalized Anxiety Disorder scale; anxiety symptom severity by score is classified as follows: 0 to 4 (asymptomatic or minimal), 5 to 10 (mild), 11 to 17 (moderate), and 18 or higher (severe).

### Program Usage Outcomes

Program usage was evaluated in the treatment cohort across a set of five program features: number of therapist consultations, number of messages sent to the therapist, number of times core lesson content was accessed, number of thoughts logged via the thought tracking tool, and number of program-related content cards viewed. As expected, there were notable correlations among these program usage factors, with Pearson coefficients ranging from 0.17 to 0.43 (Table 3).

In order to limit collinearity while evaluating the association between program usage and changes in depressive symptoms, we created a composite feature of overall depth of usage. For each usage feature, a score of 1 was assigned when activity for that specific feature was above the 25th percentile of the distribution. We then summed across each of the factors. Thus, depth-of-usage scores could range from 0 to 5, with a score of 5 indicating usage in excess of the 25th percentile of activity across the features, and a score of 0 indicating usage below the 25th percentile across the features. As noted above, the data were then resampled to adjust for the female predominance in the treatment cohort. From the resulting balanced cohort of 1430 participants, a cluster-robust logistic model controlling for age, gender, co-occurrence of anxiety (ie, baseline GAD-7 score ≥11), and baseline PHQ-8 score revealed a significant association between depth of usage and likelihood of reliable improvement in depression scores at follow-up (odds ratio [OR] 1.3, 95% CI 1.1-1.5; *P*=.002). In other words, greater usage across the platform was associated with improvement in depression symptom severity. The summary statistics for the features of program usage were as follows: mean number of therapist consultations was 7.04 (SD 3.4), mean number of messages sent to the therapist was 33.2 (SD 46.9), mean number of times core lesson content was accessed was 39.2 (SD 33), mean number of thoughts logged via the thought tracking tool was 13.6 (SD 16.5), and mean number of program-related content cards viewed was 8.35 (SD 12.4).

**Table 3:** Correlation analysis (Pearson r and 2-tailed *P* value) among the features of program usage (n=831).

Variable			Consults		Messages		Lessons opened		Thoughts logged		Content viewed
**Consults**											
	*r*	1		0.29^a^		0.26^a^		0.26^a^		0.17^a^	
	*P* value	—^b^		<.001		<.001		<.001		<.001	
**Messages**											
	*r*	0.29^a^		1		0.33^a^		0.43^a^		0.31^a^	
	*P* value	<.001		—		<.001		<.001		<.001	
**Lessons opened**											
	*r*	0.26^a^		0.33^a^		1		0.44^a^		0.35^a^	
	*P* value	<.001		<.001		—		<.001		<.001	
**Thoughts logged**											
	*r*	0.26^a^		0.43^a^		0.44^a^		1		0.28^a^	
	*P* value	<.001		<.001		<.001		—		<.001	
**Content viewed**											
	*r*	0.17^a^		0.31^a^		0.35^a^		0.28^a^		1	
	*P* value	<.001		<.001		<.001		<.001		—	

^a^The correlation is significant at a significance level of .05 (2-tailed).

^b^Not applicable.

### Exploratory Weight

We performed additional analyses to explore changes in body weight and diabetes management among participants simultaneously enrolled in the Vida CBT Program and a physical health program.

For body weight, in order to provide adequate time for meaningful clinical change, the analysis was restricted to the 595 participants with overweight or obesity (ie, BMI ≥25) who had been enrolled for at least 6 months and had logged weight at least twice in the period with the latest weight coming at least 2 months after the initial enrollment. Out of these participants, 86.6% (n=515) had a baseline BMI indicative of obesity and 13.4% (n=80) had a baseline BMI indicative of overweight. This population logged a total of 34,469 body weight values. Out of these entries, 83.17% (n=28,667) were logged using a wireless connected scale, and the remainder were entered manually by the participant. The majority of the participants (n=387, 65.0%) had logged their most recent weight in the ninth program month. The most recent weight was logged at least in the eighth program month for 78.8% (n=469) of the participants and at least in the sixth program month for 89.2% (n=531) of them.

**Figure 3:**
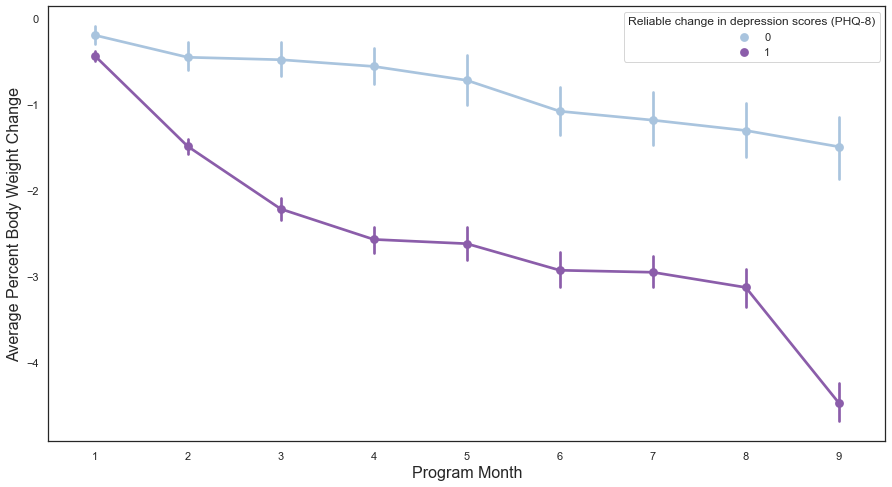
Estimated marginal means and SEs of average percent weight loss as a function of program month and reliable change in depression symptoms. A value of 1 represents reliable improvement in PHQ-8 scores and 0 represents no reliable improvement. PHQ-8: 8-item Patient Health Questionnaire.

To account for heterogeneity in the number and frequency of weight logs among participants, as well as to handle the nested structure of the data (ie, weight observations nested within participants who were nested within providers), a multilevel mixed-effects model was used to evaluate changes in body weight across participants. Fixed factors in the model were time (program month), baseline BMI category (obesity or overweight), and reliable change in depressive symptoms (1 = reliable improvement in PHQ-8 scores; 0 = no reliable improvement). Random factors included participants and providers. We observed a significant effect of program time on percent weight loss (β=–0.37, *P<*.001). Baseline weight had a small but significant inverse association with weight loss such that higher baseline weight was associated with greater percent weight loss (β=–0.01, *P*=.002). As shown in Figure 3, reliable improvement in depression symptoms at follow-up was associated with significantly greater weight loss at 9 months (β=–1.11, *P*=.002).

## Discussion

### Principal Findings

The aim of this retrospective study was to evaluate the therapist-supported, digitally delivered Vida CBT Program for the treatment of moderate depression in an adult population. The treatment cohort of 831 participants with baseline PHQ-8 scores of 10 or greater were enrolled in the digital intervention and provided at least one follow-up assessment 6 to 12 weeks into the program. We observed a significant reduction in depression scores at follow-up (mean –5.9, SD 5.2), with 74.5% of participants shifting downward in symptom severity by at least one diagnostic category. A similar pattern of reduction was observed for anxiety scores among participants who had moderate anxiety (mean –6.1, SD 5.4). Supplementary ITT analysis that included the entire cohort of 1512 participants with baseline data or last-value carryforward showed a slightly attenuated but still significant reduction in PHQ-8 scores at follow-up.

Examining the relationship between program usage and improvement in depression symptoms revealed that participants who more extensively used features across the platform had a modestly greater likelihood of reliable improvement in depression scores at follow-up (OR 1.3, 95% CI 1.1-1.5). This limited dose-response relationship was seen in the context of highly right-skewed usage data overall.

Drawn largely from a medically complex adult population, cardiometabolic conditions, particularly diabetes and obesity, were highly prevalent in this population. Given the very high burden of metabolic disease in this population and the availability of interventions for these conditions on the Vida platform, although the study was not specifically designed for this, we wanted to explore these relationships in order to prepare for future research.

Our preliminary analyses showed significantly greater reduction in body weight among participants who had a reliable improvement in depression symptoms compared to those who did not show symptom improvement at follow-up. As Figure 3 illustrates, this trend was consistent throughout the program tenure and culminated in a mean body weight change of 4.5% (SD 6.3; reliable improvement in PHQ-8 scores) versus 1.5% (SD 6.1; no reliable improvement in PHQ-8 scores) at month 9 of the program.

As with much of the literature, it is impossible to infer causation from these results, but it opens a hypothesis for future research that there may be opportunities for synergy in the treatment of co-occurring mental and metabolic disorders [37,62]. Given the massive and overlapping burden of these diseases in the US population, if such synergy could be realized through efficient, digitally delivered interventions, it would certainly be welcome.

### Comparison With Prior Work

The principal finding of improvements in depressive symptoms is consistent with the broader body of research on digitally delivered CBT programs for the treatment of mild to moderate depression and anxiety [9]. This finding reinforces, in a larger population, similar results previously published from the Vida CBT Program [23].

The relationship between program usage and outcomes in digital interventions, broadly, and DMHIs, in particular, remains a rich area of research. While this study was not designed to evaluate this, our results are consistent with several similar studies suggesting a limited dose-response phenomenon. That is, greater usage seems to correlate with improved outcomes but may not do so monotonically, as participants self-regulate their usage to their needs, needs that are difficult for a researcher to observe with any meaningful precision [18,63-66].

Throughout the literature, interrelations among treatments for comorbid mental and physical disorders have been noted before. The direction of causality is unclear and, indeed, may be expected to run in both directions; that is, depression and anxiety may contribute to obesity and diabetes just as much as the reverse is true. In some cases, other factors may be driving both [62,67-69]. Preliminary research in this vein has, like this study, shown some hint of synergistic treatment effects in traditional care settings [70-72].

### Limitations

This study had several important limitations. The lack of a control group and the retrospective design prevents drawing any causal inferences. Missing follow-up data was also a challenge, with data unavailable for 45.0% of the overall cohort (Table 1). We attempted to mitigate this limitation with an ITT analysis using baseline data or last-value carryforward as appropriate. While this still showed a significant relationship, the improvement in depression scores was weaker when accounting for those participants.

The gender imbalance across the study cohort was notable, with 83.8% females overall and 86.0% in the treatment group. At the overall level, this is a well-characterized phenomenon of care use, particularly for mental health, in North America and Europe. Despite seemingly comparable prevalence of mental distress by gender, ratios of 2 women for every man seeking care are not unusual in the literature [73,74]. The US National Institute of Mental Health has even sponsored campaigns to address barriers to males seeking mental health care, and there is literature on this phenomenon’s underlying causes [56,75]. In this study, there was also a small but significant increase in the proportion of females in the treatment group. It may have been that whatever phenomenon inhibits males from seeking care, in general, also made males in this study less likely to follow up with treatment and assessment. This and the difference in age between the two groups could represent some self-selection of participants into the treatment group who were more likely to improve. While the ITT analysis attempts to adjust for this, some bias remains a possibility. More broadly, the limited uptake of the Vida intervention by males may limit its generalizability in a larger population.

This study was not specifically designed to investigate changes in body weight and glycemic control as related to improvements in depression and was underpowered to detect a change in HbA_1c_. Future research is certainly needed to explore these potential relationships. Causality in any such relationship, should it bear out, could take any possible form and would need careful study to dissect.

### Conclusions

This study provides further evidence that CBT-based DMHIs can be effective tools in treating symptoms of depression and anxiety. Improvements by whole diagnostic categories were common in this cohort. Usage of the platform correlated with improved outcomes in a pattern consistent with participants self-regulating usage to their individual needs. Furthermore, this was seen in a population with a very high prevalence of co-occurring physical disorders. More research is required to determine if there may be opportunities for synergistic treatment of mental and physical disorders through a similar modality.

## Abbreviations

CBT: cognitive behavioral therapy
DMHI: digital mental health intervention
GAD-7: 7-item Generalized Anxiety Disorder scale
HbA_1c_: hemoglobin A_1c_
ITT: intention-to-treat
OR: odds ratio
PHQ-8: 8-item Patient Health Questionnaire
RCI: reliable change index
